# Temperature Effects in Packaged RF MEMS Switches with Optimized Gold Electroplating Process

**DOI:** 10.3390/mi15091085

**Published:** 2024-08-28

**Authors:** Lifeng Wang, Lili Jiang, Ning Ma, Xiaodong Huang

**Affiliations:** 1 Key Laboratory of MEMS of the Ministry of Education, School of Electronic Science & Engineering, Southeast University, Nanjing 210096, China; 230199026@seu.edu.cn (L.J.); 13260933933@163.com (N.M.); xdhuang@seu.edu.cn (X.H.); 2 Nanjing Electronic Devices Institute, Nanjing 210016, China

**Keywords:** RF MEMS switch, gold electroplating process, temperature effects, pull-in voltage, lifetime

## Abstract

Due to its excellent electrical performance, mechanical reliability, and thermal stability, electroplated gold is still the most commonly used material for movable beams in RF MEMS switches. This paper investigates the influence of process conditions on the quality and growth rate of gold electroplating, and the optimized process parameters for the gold electroplating process are obtained. The characterization of the optimized electroplated gold layer shows that it has small surface roughness and excellent thermal stability. With this optimized gold electroplating process, the RF MEMS switches are fabricated and hermetic packaged. In order to obtain the temperature environment adaptability of the packaged switch, the influence of working temperature is studied. The temperature effects on mechanical performance (includes pull-in voltage and lifetime) and RF performance (includes insertion loss and isolation) are revealed.

## 1. Introduction

An RF switch is an important component in the fields of wireless communication and high-frequency circuits. An RF MEMS switch is a type of RF switch made by MEMS technology, and is one of the most successful devices in the RF field using MEMS technology [1]. Compared with traditional solid-state RF switches, RF MEMS switches use mechanical structures to control the on/off state of signals, which gives them advantages in terms of insertion loss, isolation, linearity, etc. [2]. RF MEMS switches have broad application prospects in microwave-integrated circuits and wireless communications, such as phase shifters, switch-type filters, switch arrays, radar TR modules, and satellite communication [3].

From the aspect of signal processing, RF MEMS switches are divided into resistive and capacitive types; from the aspect of electrical connection, RF MEMS switches are divided into series and parallel types. Therefore, RF MEMS switches are mainly divided into four types: series resistive, parallel resistive, series capacitive, and parallel capacitive [4,5,6,7]. Based on these four basic types, researchers continuously improve the performance of switches by structural design. As important as structural design, researchers have also attempted to improve the performance of switches from the aspects of fabrication processes and materials. Kumar et al. conducted optimization research on the process parameters of the photoresist sacrificial layer of the switch. Based on the optimized photolithography process parameters, high-performance switches are obtained [8]. Persano et al. studied the effects of different movable beam sizes and process parameters on the RF performance of switches [9]. Bajpai et al. improved the isolation performance of switches by reducing the surface roughness of floating metal films [10]. By developing a double sacrificial layer process, Lee et al. provided a new method to control the membrane deformation and composite stiffness of the movable beam [11]. Fruehling et al. developed an SOI-based switch fabrication process. By using single-crystal silicon as the movable beam, the switch achieves a very short switching time and good mechanical stability [12]. By using standard 0.35 mm CMOS technology, Fouladi et al. proposed an RF MEMS switch with a movable beam made of Al/SiO_2_ composite material. Although the RF performance is not outstanding, its biggest advantage is that it can be mass-produced using mature CMOS technology [13]. Kurmendra et al. summarized the material selection and the fabrication technology of RF MEMS switches. Due to its excellent electrical performance, mechanical reliability, and thermal stability, Au is still the most commonly used movable beam material, followed by Al and Cu. The most commonly used manufacturing process for switches is the surface sacrificial layer process, followed by the bulk process. In addition, switch processes compatible with the CMOS process are also receiving increasing attention [14].

After fabrication, switches are usually hermetically packaged to improve their reliability. Packages can isolate dust and moisture in the environment. However, the temperature variation still may have an influence on the switches. To investigate the thermal effects on the reliability of RF MEMS switches, a current density model and a thermal model are coupled to calculate the current density, heat loss, and temperature rise of the switch [15]. Mulloni et al. measured and discussed the variation of actuation voltage with temperature for clamped–clamped switches and cantilever switches. Experimental results indicate that the performances of cantilever switches are less influenced by the temperature [16]. They also established a lifetime model for the switches and used temperature as an acceleration factor to predict the lifetime of the switches [17]. Yuan et al. characterized the temperature acceleration of dielectric charging effects in RF mems switches. A charging model is provided to predict the amount of charging under different temperatures [18]. Gong et al. studied the temperature effect on the mechanical and RF performance of the switch. However, they mainly focused on cryogenic temperatures that are below room temperature [19].

Based on our previous work [20,21], this paper investigates the influence of process conditions on the quality and growth rate of gold electroplating. With the optimized gold electroplating process, the RF MEMS switches are fabricated and hermetic packaged. In order to obtain the temperature environment adaptability of the packaged switch, the influence of working temperature on the mechanical and RF performance of the switch is studied. The main content of this paper is organized as follows, Section 2 designs the switch structure and the hermetic packaging; Section 3 studies the optimization of the gold electroplating process and tests the temperature stability of the gold beam; Section 4 tests the effects of temperature on the mechanical and RF performance of the switch; and, finally, some conclusions are given.

## 2. Switch Design

**Figure 1:**
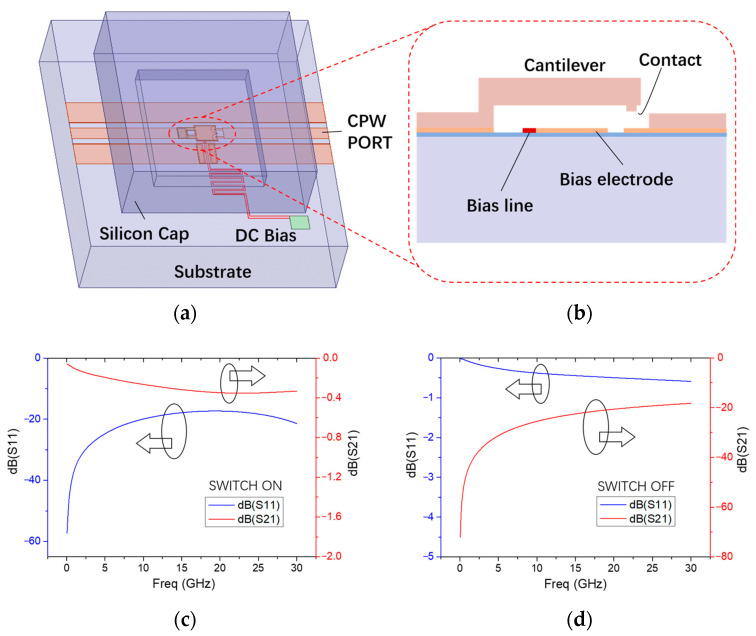
RF MEMS switch design: (**a**) Schematic diagram of the switch; (**b**) The cross-sectional view of the movable part; (**c**) Simulated ON state RF performances of the designed switch witch package; (**d**) Simulated OFF state RF performances of the designed switch with the package.

Figure 1a shows a schematic diagram of a series of resistive RF MEMS switches designed with a hermetic package. The complete switch structure consists of substrate, coplanar waveguide (CPW) transmission line, movable cantilever beam, DC bias, and package cap. The substrate uses high-resistance silicon (HRS) with a resistivity greater than 6000 Ω·cm and a thickness of 400 mm. The material of CPW and cantilever beam is thick electroplated Au, which can reduce the RF loss of the switch as well as enhance the mechanical strength of the movable beam. The DC bias line is made of TiWSi with medium resistance and is designed into a serpentine shape for RF/DC isolation. The package cap also uses high-resistance silicon, the same as the substrate, to reduce RF loss.

Figure 1b is a cross-sectional view of the movable part of the switch. One side of the movable beam is connected to the anchor, and the other side has a contact structure. The DC bias electrode is located directly below the movable beam. When a sufficiently large driven voltage is applied to the bias electrode, the cantilever beam moves downward and contacts the transmission line, which is called the ON state of the switch; when the driven voltage is removed, the cantilever beam rebounds and disconnects from the transmission line, which is called the OFF state of the switch. From Figure 1b, it can be seen that there is no insulating dielectric layer between the bias electrode and the movable beam.

The key parameters of the switch are listed in Table 1. The RF performances are simulated in finite element software HFSS 15.0. Figure 1c,d show the simulated ON state and OFF state RF performances of the designed switch witch package, respectively. The simulated insertion loss of the switch with the package is −0.33 dB @ 30 GHz, and the isolation is −18.33 dB @ 30 GHz. The mechanical performance of the designed switch is simulated in CoventorWare v2012. The simulated pull-in voltage is 52 V.

## 3. Fabrication

### 3.1. Optimization of Gold Electroplating Process

Among the fabrication processes of the switches, the gold electroplating process plays a decisive role in the mechanical as well as the RF properties of the switch, including the pull-in voltage, temperature stability, insertion loss, and lifetime. In order to achieve high-quality electroplating of gold, the electroplating parameters are studied and optimized.

Commonly used electroplating methods include DC electroplating and pulse electroplating. Compared with DC electroplating, pulse electroplating can effectively reduce the difference in deposition rate and diffusion rate, thus improving the growth of the grain. The electroplating solution is based on potassium aurous cyanide.

**Figure 2:**
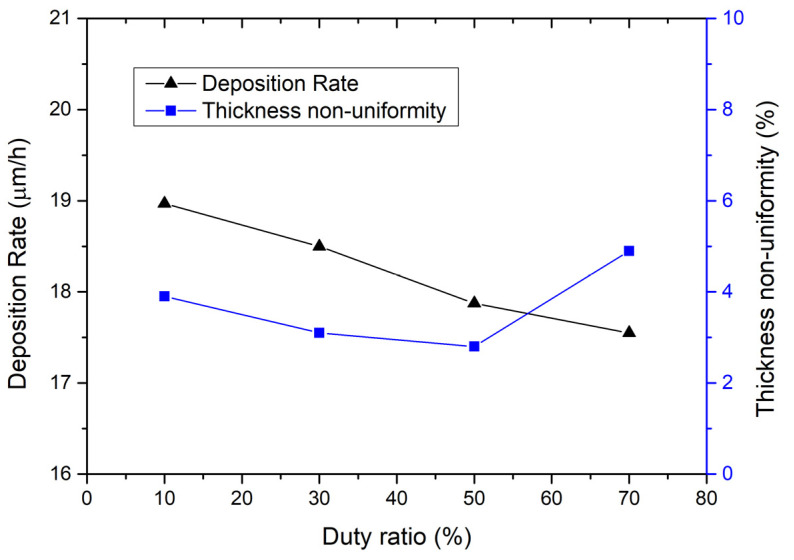
On the left is the relationship between deposition rate and duty ratio; on the right is the relationship between thickness non-uniformity and duty ratio. The electroplating condition frequency is 1 kHz, current density is 0.5 A/dm^2^, temperature is 50 °C, and the flow rate is 20 L/min.

The effect of the duty ratio of the pulse on the electroplating process is measured and shown in Figure 2. The calculation method of the thickness non-uniformity used here is (maximum thickness − minimum thickness)/(2 × average thickness)) × 100%. As can be seen from Figure 2, the deposition rate curve shows a trend of decreasing with the duty ratio. When the duty ratio increases from 10% to 70%, the deposition rate decreases from 18.97 μm/h to 17.55 μm/h. The reason is that, as the duty ratio increases, the current shutdown time gradually decreases, and the solution does not have enough time to restore the metal ions near the cathode to their initial concentration, thereby reducing the current efficiency and causing a slight decrease in the deposition rate of the coating metal. However, the thickness non-uniformity curve in Figure 2 shows a different trend, i.e., it decreases first and then increases with the duty ratio. When the duty ratio is 50%, the thickness non-uniformity reaches a minimum of 2.8%. Therefore, it is recommended to set the duty ratio of the pulse to 50%.

**Figure 3:**
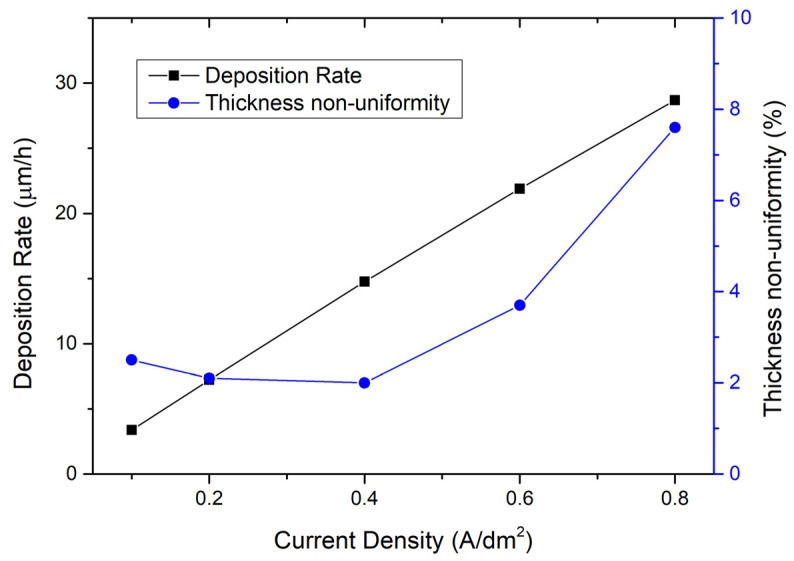
On the left is the relationship between deposition rate and current density; on the right is the relationship between thickness non-uniformity and current density. The electroplating condition frequency is 1 kHz, duty ratio is 50%, temperature is 50 °C, and the flow rate is 20 L/min.

Then, the influence of the current density on the electroplating process is studied. Figure 3 shows the measured curve of deposition rate with current density. It can be seen that, in the range of 0.1 A/dm^2^ to 0.8 A/dm^2^, the deposition rate increases linearly with the increase in current density. The thickness non-uniformity curve in Figure 3 shows that, when the current density is between 0.1 A/dm^2^ and 0.6 A/dm^2^, the electroplating thickness non-uniformity is relatively good, i.e., between 2.0% and 3.7%; and when the current density reaches 0.8 A/dm^2^, the thickness non-uniformity deteriorates significantly, to 7.6%. This indicates that an excessively high current density will lead to poor electroplating non-uniformity. Compromising between the electroplating rate and thickness non-uniformity, the recommended current density is from 0.4 A/dm^2^ to 0.6 A/dm^2^.

**Figure 4:**
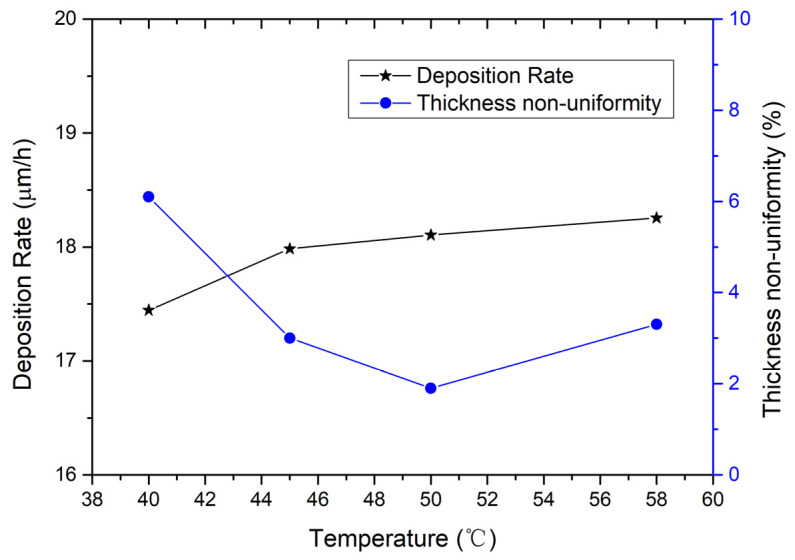
On the left is the relationship between deposition rate and temperature; on the right is the relationship between thickness non-uniformity and temperature. The electroplating condition frequency is 1 kHz, duty ratio is 50%, current density is 0.5 A/dm^2^, and the flow rate is 20 L/min.

The impact of the temperature of the plating solution on the deposition rate and thickness non-uniformity is also investigated. In Figure 4, the left side shows the relationship between deposition rate and temperature. When the temperature increases from 45 °C to 58 °C, the deposition rate increases slightly from 17.99 µm/h to 18.26 µm/h; and when the temperature drops to 40 °C, the deposition rate decreases to 17.44 µm/h. The relationship between thickness non-uniformity and temperature is also shown in Figure 4. From 45 °C to 58 °C, the electroplating exhibits good thickness non-uniformity between 1.9% and 3.3%; when the temperature lowers to 40 °C, the thickness non-uniformity worsens, rising to 6.1%. According to the experimental results, the recommended temperature of the plating solution is 50 ± 5 °C.

In addition, the influences of pulse frequency and solution flow rate on the deposition rate and quality of gold electroplating were also studied. The optimized parameters of the gold electroplating process are shown in Table 2.

### 3.2. Characterization of the Optimized Electroplated Gold

The characterization of the optimized electroplated gold mainly includes a surface roughness scan and a profile scan.

**Figure 5:**
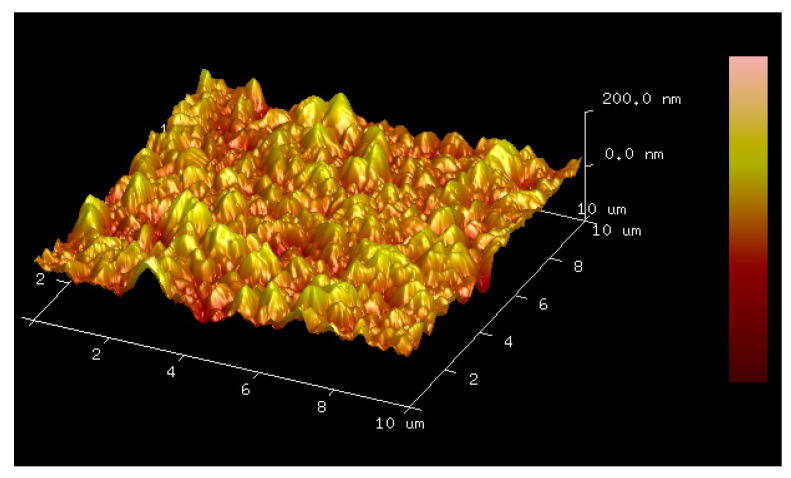
The surface roughness of the electroplated gold tested by AFM.

Firstly, AFM is used to test the surface roughness of the electroplated gold structure, and the test results are shown in Figure 5. It can be seen that the surface grains of electroplated gold are fine and uniform. The measured Roughness Average (Ra) is 46 nm, RMS Roughness (Rq) is 57 nm, Maximum Profile Valley Depth (Rv) is 138 nm, and Maximum Height of the Profile (Rt) is 286 nm.

**Figure 6:**
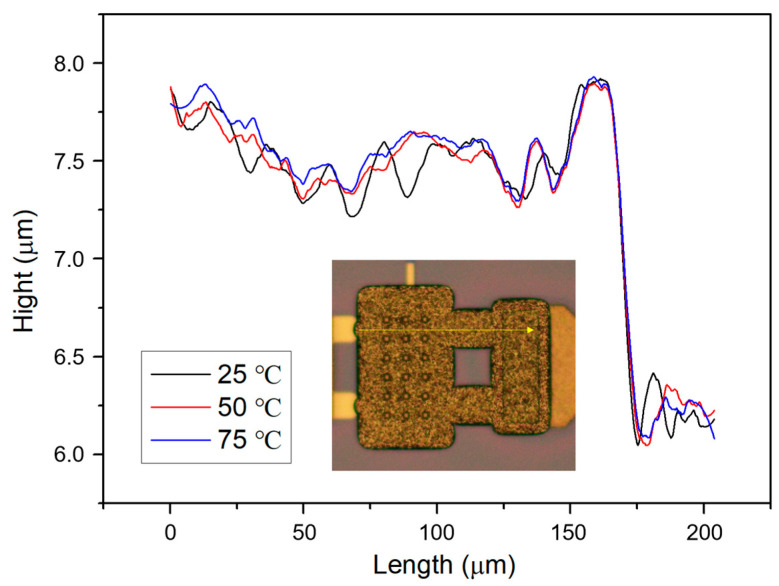
Profile scans of the released electroplated gold beam. The inset displays the released gold beam and profile scanning path.

A profile scan is conducted on the released electroplated gold beam, and the temperature stability of the beam profile is tested at different temperatures. The test results are shown in Figure 6, in which the inset displays the released gold beam and profile scanning path. Firstly, from the scanned profile, it can be seen that the gold beam does not show significant warping after release, indicating that the internal stress of the electroplated gold layer is relatively low. Secondly, at the range of 25 to 75 °C, the profiles of the cantilever show very small deviates, which means the electroplated gold layer has excellent thermal stability.

**Figure 7:**
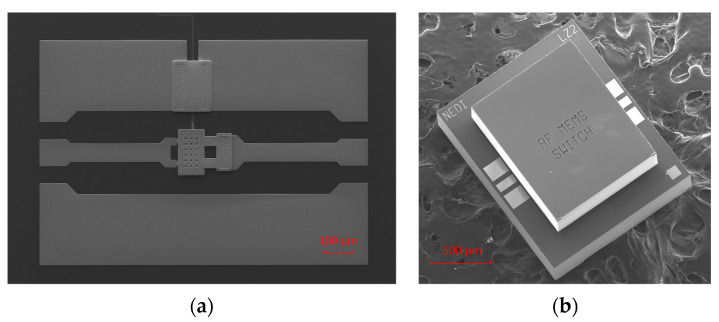
(**a**) SEM image of the fabricated switch. (**b**) SEM image of the switch with a hermetic package.

The process flow of the switch as well as its hermetic package method can refer to our previous work [20]. The SEM of the fabricated RF MEMS switch using the optimized gold electroplating process above is shown in Figure 7a, and the SEM image of the hermetic packaged switch with an HRS cap is shown in Figure 7b.

## 4. Measurements and Discussion

### 4.1. Temperature Effect on Mechanical Performance

**Figure 8:**
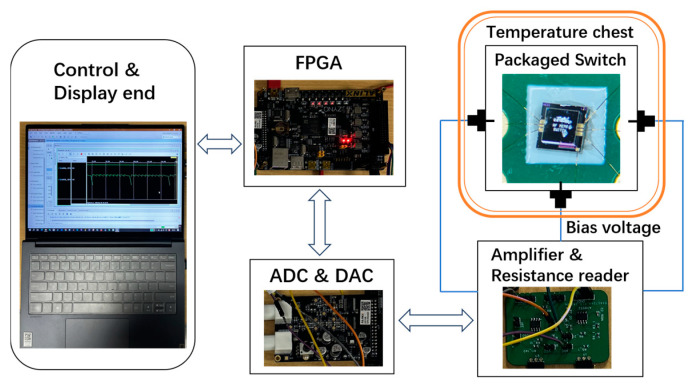
An automatic testing system for pull-in voltage and lifetime test of the switch.

Firstly, in order to facilitate continuous and automatic testing of the pull-in voltage and lifetime of the switch, an automatic testing system was designed. The testing platform includes FPGA modules, high-speed 8-bit AD/DA modules, amplification and resistance reading circuits, and control and display end. The connections between each module are shown in Figure 8. The testing system uses the FPGA module to control the DAC to generate periodic signals with adjustable frequency and waveform. Then, the signal is amplified by the amplification circuit to control the on/off state of the switch. At the same time, the resistance reading circuit collects the series resistance of the switch and transmits it to the FPGA module through ADC. The FPGA can generate sawtooth waves or square waves, which are used for pull-in voltage measurement or lifetime testing, respectively. During the pull-in voltage measurement phase, the amplification circuit outputs sawtooth waves. When the switch is pulled in, the values of the DAC and ADC at the moment correspond to the pull-in voltage and on-state resistance of the switch, respectively. During the lifetime testing phase, the amplification circuit outputs square waves. When the on-state resistance suddenly increases or remains unchanged at low resistance, it indicates that the switch has failed. In order to investigate the effect of temperature on the performance, the temperature chest of Cascade SUMMIT-12000B is used to control the temperature environment of the packaged switch.

**Figure 9:**
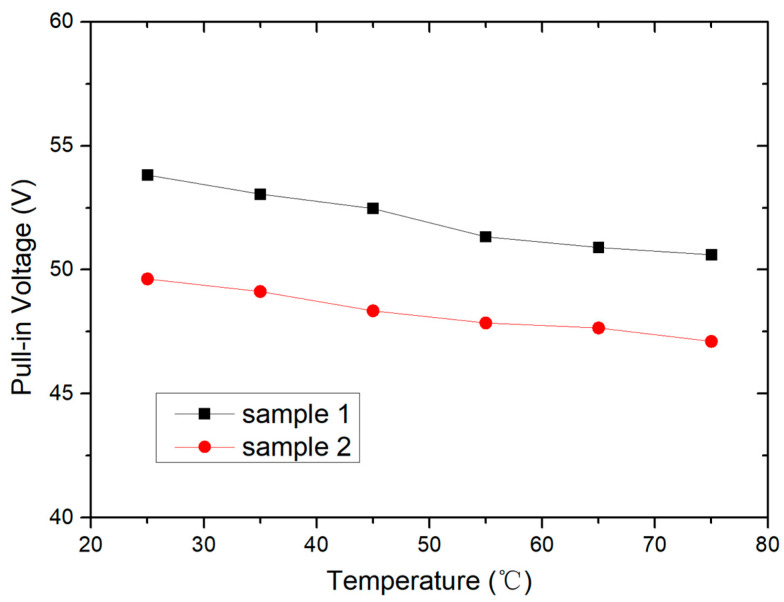
Temperature effect on the pull-in voltage of the packaged switches.

At room temperature, the pull-in voltage range of the fabricated switches is measured to be 45–60 V, and the on-state resistance R_on_ is between 1.5 and 2.5 ohms. Then, the temperature effect on pull-in voltage is tested, and the test results are shown in Figure 9. The testing interval for each temperature point is 1 h to ensure the stability of the temperature environment and switch status. When testing at a certain temperature point, the testing system continuously generates five sawtooth waves, and the average value of the five measured pull-in voltages is obtained. It can be seen that in the range of 25 to 75 °C, the switching voltage decreases nearly linearly with temperature.

In order to analyze how temperature variation affects the pull-in voltage, the theoretical formula of the pull-in voltage *V_p_* of the switch is given as [1]

(1) $$V_{p}=\sqrt{\frac{8k{g_{0}}^{3}}{27\varepsilon_{0}A}}$$

in which *k* is the elastic coefficient of the movable beam, *g*_0_ is the gap between the movable beam and the bias electrode, and *A* is the overlapping area between the movable beam and the bias electrode. In the above equation, the parameters that may vary with temperature include the elastic coefficient *k*, gap *g*_0_, and area *A*. Among them, in the range of 25 to 75 °C, the change in area A can be ignored. The variation in gap *g*_0_ is related to the deformation of the movable beam. According to the profile scan results in Figure 6, the deformation of the movable beam in the range of 25 to 75 °C can be ignored. The elastic coefficient k is determined by the geometric dimensions and Young’s modulus of the movable beam. Among them, the variation in geometric dimensions with temperature can be ignored. Therefore, the temperature effect of Young’s modulus of electroplated gold materials is the main reason for the variation of the switch pull-in voltage with temperature [22].

**Figure 10:**
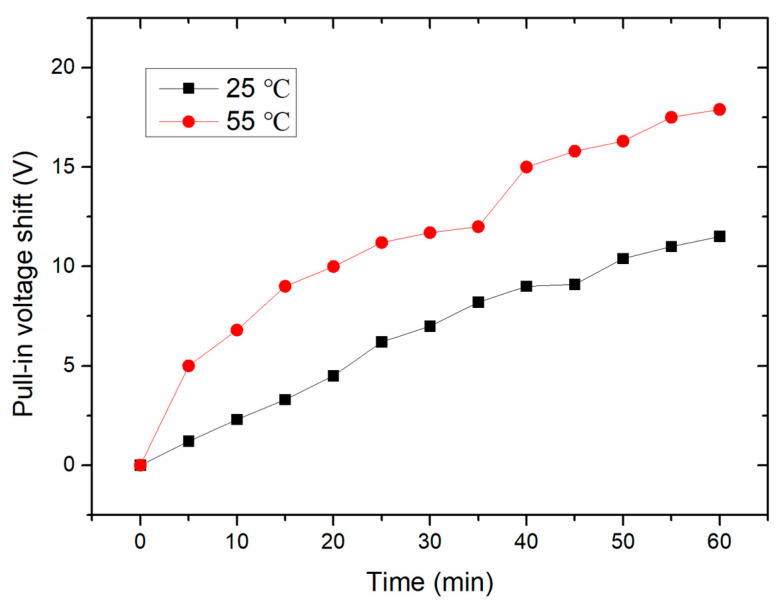
The pull-in voltage shift with the operation time at different temperatures.

Next, under different temperatures, the pull-in voltage shift of the switch during 1 h continuous operation is measured. The actuation voltage used here is a 1 kHz, 65 V square wave. A sawtooth wave is generated every 5 min to obtain the pull-in voltage of the switch. Figure 10 shows the pull-in voltage shift with the operation time at different temperatures. Firstly, the pull-in voltage continuously increases with the operation time of the switch, which is consistent with the dielectric charging effect of RF MEMS switches [23,24]. Secondly, the shift of the pull-in voltage significantly increases with the increase in environment temperature, which is consistent with the test results in Ref [18].

**Figure 11:**
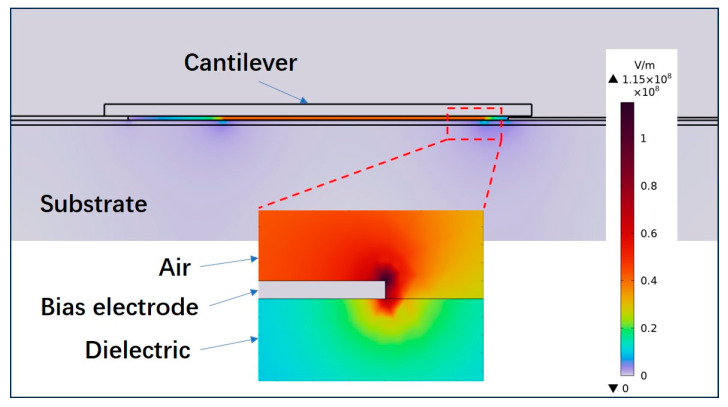
Electric field distribution of the switch under DC bias voltage.

The phenomenon of charge injection is widely used in ohmic contact switches and capacitive switches with a dielectric layer between the driven electrodes [25,26]. The switch designed here has no dielectric layer between the driven electrodes, but the test results show that the dielectric charging still happens. In order to analyze the source of charge injection, COMSOL is used to perform electrostatic simulation on the switch, as shown in Figure 11. From the simulation results, most of the electric field exists in the air between the cantilever beam and the bias electrode. But there are also large electric fields of 60–80 MV/m around and underneath the bias electrode. These large electric fields are sufficient to inject charges into the dielectric layer below the bias electrode.

**Figure 12:**
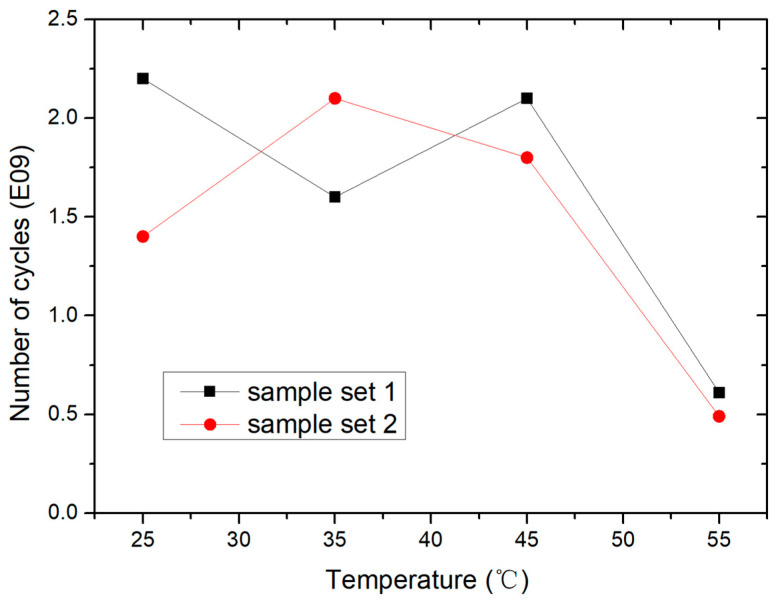
The lifetime of the switches at different temperatures.

After that, the lifetime of the switch is tested at different temperatures. The driven signal used for the lifetime test is a 10 kHz, 80 V square wave. The lifetime test results of the switch at different temperatures are shown in Figure 12. From the test results, it can be seen that the switch lifetime is about 2 × 10^9^ within the range of 25–45 °C. In this temperature range, the effect of temperature on switch lifetime is not significant. When the temperature reaches 55 °C, the lifetime of the switch decreases significantly to 0.5 × 10^9^, and the switches all failed due to unchanged high resistance. The reason may be the acceleration effect of temperature on charge injection.

### 4.2. Temperature Effect on RF Performance

**Figure 13:**
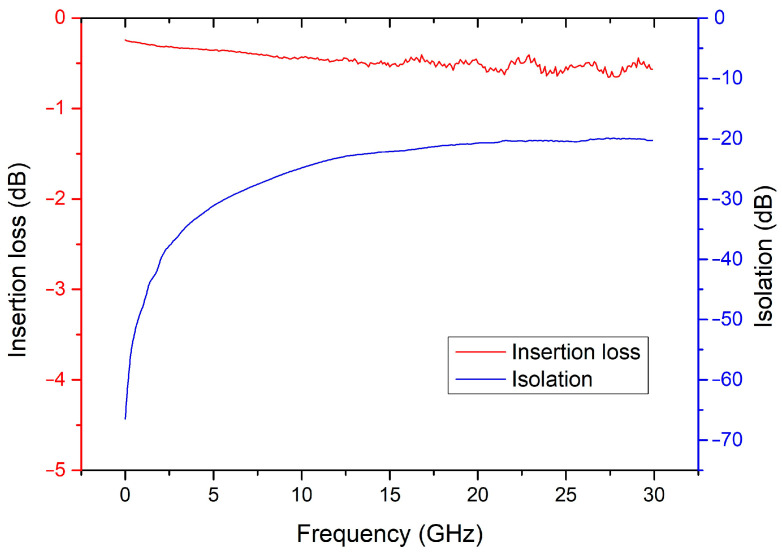
The insertion loss and isolation curves of the packaged switch measured at room temperature.

Figure 13 shows the insertion loss and isolation curves of the packaged switch measured at room temperature. The measured insertion losses of the switch are −0.3 dB @ 10 GHz and −0.6 dB @ 30 GHz; the measured isolations are −25.2 dB @ 10 GHz and −20.1 dB @ 30 GHz.

**Figure 14:**
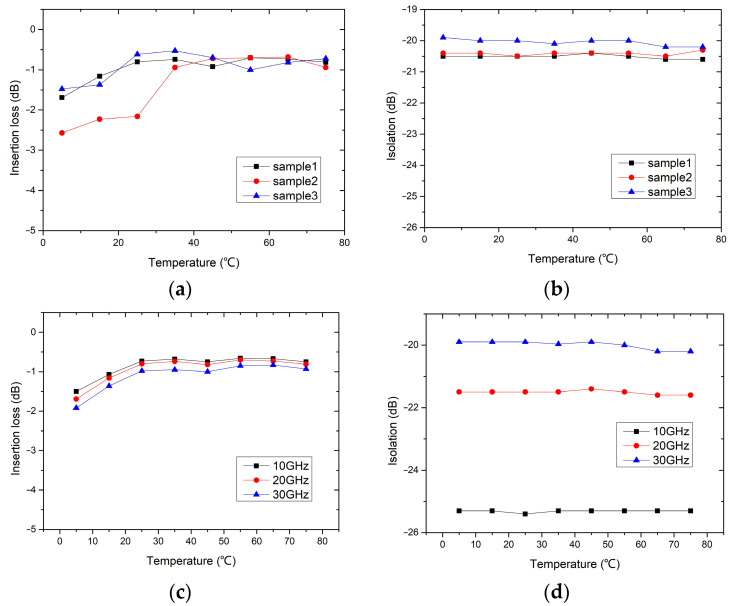
The RF performance variation of the switches working at different temperature environments: (**a**) The insertion loss variation of three switch samples versus temperature @ 30 GHz; (**b**) The isolation variation of three switch samples versus temperature @ 30 GHz; (**c**) The insertion loss variation at different operation frequencies versus temperature; (**d**) The isolation variation at different operation frequencies versus temperature.

The RF performance variation of the switches working at different temperature environments is tested and shown in Figure 14. The working temperature is raised from 5 to 75 °C in steps of 10 °C, and the RF performance of the switches is read after each temperature point has been stabilized for 1 h. The insertion losses of three switch samples versus working temperature are shown in Figure 14a. It can be seen that the insertion losses of the switches decrease with the increasing temperature. The reason may be that higher temperature softens the contact material and improves the contact effect. The isolation of three switch samples versus working temperature is shown in Figure 14b. The isolation of the switch is almost unaffected by the working temperature. Since the isolation of the switch is related to the position of the movable beam, the results in Figure 6 indicate that temperature has almost no effect on the position of the movable beam. This is consistent with the test results shown in Figure 14b.

Finally, Figure 14c,d show the temperature effects on insertion loss and isolation of the switch at different operating frequencies, respectively. It can be seen that the trend of RF performance changing with temperature is consistent at different operating frequencies.

## 5. Conclusions

In summary, the influence of process conditions on the quality and growth rate of gold electroplating is investigated, and the optimized process parameters for the gold electroplating process are obtained. The characterization of the optimized electroplated gold layer shows that it has small surface roughness and excellent thermal stability. With this optimized gold electroplating process, the RF MEMS switches are fabricated and hermetic packaged, and the temperature effects on the mechanical and RF performance of the packaged switch are revealed.

## Figures and Tables

**Table 1:** The key parameters of the switch.

Parameter	Value
GSG	40/80/40 μm
Beam thickness	5 μm
Beam spring size	40 × 40 μm
Beam electrode size	100 × 160 μm
Electrostatic gap	1.8 μm
Contact thickness	0.5 μm
Cavity depth of silicon cap	100 μm

**Table 2:** The optimized parameters of gold electroplating.

Parameter	Value
Current density	0.4~0.6 A/dm^2^
Temperature	50 ± 5 °C
Frequency	1 kHz
Duty ratio	50%
Flow rate	10~30 L/min

## Data Availability

The original contributions presented in the study are included in the article, further inquiries can be directed to the corresponding author.
