# Correction: Wang et al. Temperature Effects in Packaged RF MEMS Switches with Optimized Gold Electroplating Process. *Micromachines* 2024, *15*, 1085

**DOI:** 10.3390/mi16101161

**Published:** 2025-10-14

**Authors:** Lifeng Wang, Lili Jiang, Ning Ma, Xiaodong Huang

**Affiliations:** 1 Key Laboratory of MEMS of the Ministry of Education, School of Electronic Science & Engineering, Southeast University, Nanjing 210096, China; 230199026@seu.edu.cn (L.J.); 13260933933@163.com (N.M.); xdhuang@seu.edu.cn (X.H.); 2 Nanjing Electronic Devices Institute, Nanjing 210016, China

## 1. Errors in Figures 2–4

It was found that the temperature control of the electroplating station in the previously published paper [1] is inaccurate. After calibrating the temperature control of the electroplating station, we conducted a new electroplating experiment and found that there were errors or deviations in the electroplating data in Figures 2–4. Due to these errors, the inset photos in Figures 2–4 were also incorrect. As an alternative, we adopted the thickness non-uniformity to characterize the quality of the electroplated film, which is more statistically significant and more accurate. The calculation method of the thickness non-uniformity used here is ((maximum thickness − minimum thickness)/(2 × average thickness)) × 100%.

Therefore, the corrected Figure 2, Figure 3 and Figure 4 are as follows.

## 2. Text Correction

The text related to Figures 2–4 is revised as follows.

A correction has been made to Section 3, Sub-section 3.1, Paragraph 3:

The effect of the duty ratio of the pulse on the electroplating process is measured and shown in Figure 2. The calculation method of the thickness non-uniformity used here is (maximum thickness − minimum thickness)/(2 × average thickness)) × 100%. As can be seen from Figure 2, the deposition rate curve shows a trend of decreasing with the duty ratio. When the duty ratio increases from 10% to 70%, the deposition rate decreases from 18.97 μm/h to 17.55 μm/h. The reason is that, as the duty ratio increases, the current shutdown time gradually decreases, and the solution does not have enough time to restore the metal ions near the cathode to their initial concentration, thereby reducing the current efficiency and causing a slight decrease in the deposition rate of the coating metal. However, the thickness non-uniformity curve in Figure 2 shows a different trend, i.e., it decreases first and then increases with the duty ratio. When the duty ratio is 50%, the thickness non-uniformity reaches a minimum of 2.8%. Therefore, it is recommended to set the duty ratio of the pulse to 50%.

A correction has been made to Section 3, Sub-section 3.1, Paragraph 4:

Then, the influence of the current density on the electroplating process is studied. Figure 3 shows the measured curve of deposition rate with current density. It can be seen that, in the range of 0.1 A/dm^2^ to 0.8 A/dm^2^, the deposition rate increases linearly with the increase in current density. The thickness non-uniformity curve in Figure 3 shows that, when the current density is between 0.1 A/dm^2^ and 0.6 A/dm^2^, the electroplating thickness non-uniformity is relatively good, i.e., between 2.0% and 3.7%; and when the current density reaches 0.8 A/dm^2^, the thickness non-uniformity deteriorates significantly, to 7.6%. This indicates that an excessively high current density will lead to poor electroplating non-uniformity. Compromising between the electroplating rate and thickness non-uniformity, the recommended current density is from 0.4 A/dm^2^ to 0.6 A/dm^2^.

A correction has been made to Section 3, Sub-section 3.1, Paragraph 5:

The impact of the temperature of the plating solution on the deposition rate and thickness non-uniformity is also investigated. In Figure 4, the left side shows the relationship between deposition rate and temperature. When the temperature increases from 45 °C to 58 °C, the deposition rate increases slightly from 17.99 µm/h to 18.26 µm/h; and when the temperature drops to 40 °C, the deposition rate decreases to 17.44 µm/h. The relationship between thickness non-uniformity and temperature is also shown in Figure 4. From 45 °C to 58 °C, the electroplating exhibits good thickness non-uniformity between 1.9% and 3.3%; when the temperature lowers to 40 °C, the thickness non-uniformity worsens, rising to 6.1%. According to the experimental results, the recommended temperature of the plating solution is 50 ± 5 °C.

A correction has been made to Section 3, Sub-section 3.1, Paragraph 6:

In addition, the influences of pulse frequency and solution flow rate on the deposition rate and quality of gold electroplating were also studied. The optimized parameters of the gold electroplating process are shown in Table 2.

This correction does not invalidate the other reported measurement results or the conclusions drawn from the experimental results. The authors apologize for any inconvenience this change may have caused. This correction was approved by the Academic Editor. The original publication has also been updated.

## Figures and Tables

**Figure 2:**
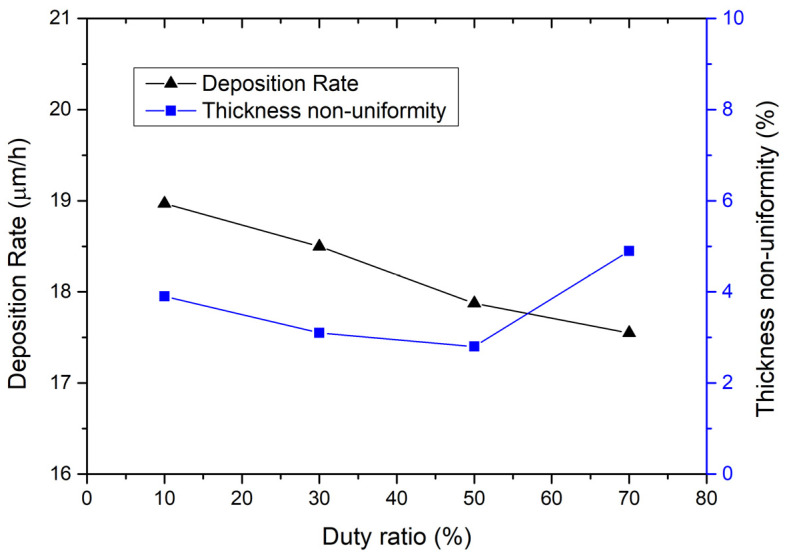
On the left is the relationship between deposition rate and duty ratio; on the right is the relationship between thickness non-uniformity and duty ratio. The electroplating condition frequency is 1 kHz, current density is 0.5 A/dm^2^, temperature is 50 °C, and the flow rate is 20 L/min.

**Figure 3:**
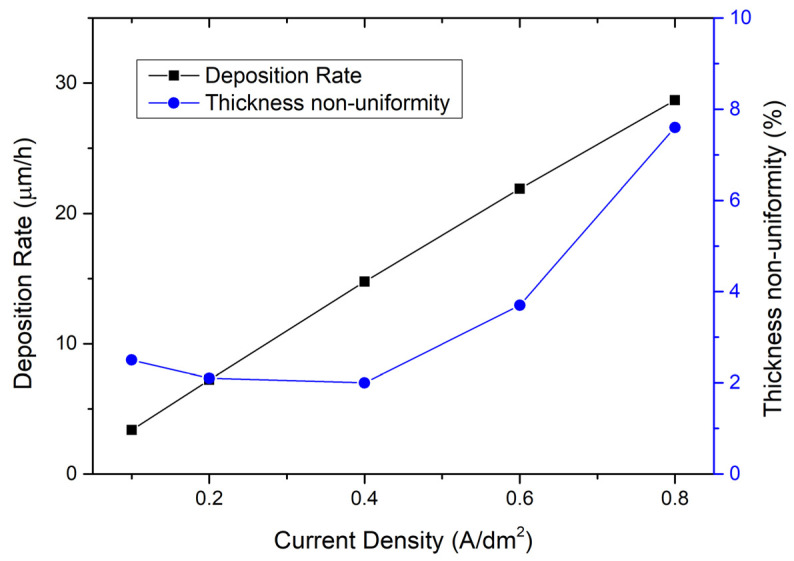
On the left is the relationship between deposition rate and current density; on the right is the relationship between thickness non-uniformity and current density. The electroplating condition frequency is 1 kHz, duty ratio is 50%, temperature is 50 °C, and the flow rate is 20 L/min.

**Figure 4:**
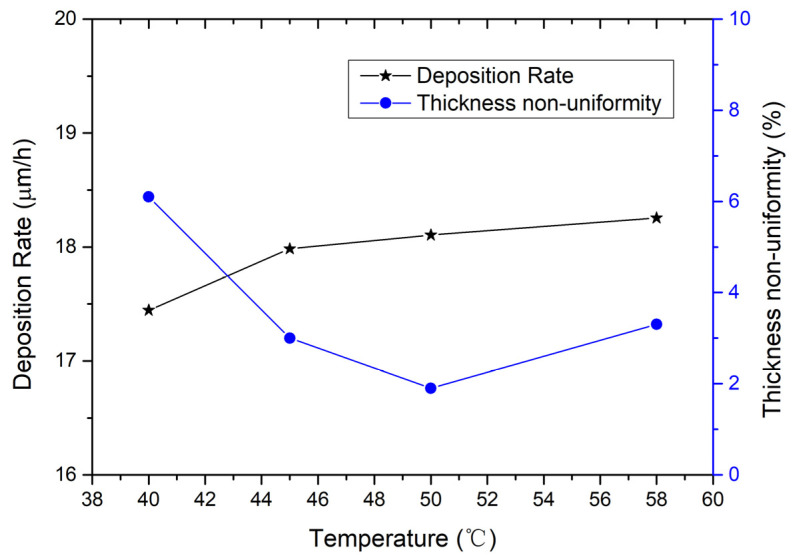
On the left is the relationship between deposition rate and temperature; on the right is the relationship between thickness non-uniformity and temperature. The electroplating condition frequency is 1 kHz, duty ratio is 50%, current density is 0.5 A/dm^2^, and the flow rate is 20 L/min.
